# Working toward improved monitoring of *Cryptosporidium* and *Giardia* (oo)cysts in water samples: testing alternatives to elution and immunomagnetic separation from USEPA Method 1623.1

**DOI:** 10.1186/s13104-022-06118-9

**Published:** 2022-07-15

**Authors:** Marie-Stéphanie Fradette, Steve J. Charette

**Affiliations:** 1 Institut de Biologie Intégrative et des Systèmes (IBIS), Laval University, 1030, avenue de la Médecine, Québec City, Québec G1V 0A6 Canada; 2 Département de Biochimie, de Microbiologie et de Bio-Informatique, Faculté des sciences et de génie, Laval University, 1045, avenue de la Médecine, Québec City, Québec G1V 0A6 Canada; 3 Centre de Recherche en Aménagement et Développement du Territoire (CRAD), 2325, allée Des Bibliothèques, Laval University, Québec City, Québec G1V 0A6 Canada; 4 Centre de Recherche de l’Institut Universitaire de Cardiologie et de Pneumologie de Québec, 2725, chemin Sainte-Foy, Québec City, Québec G1V 4G5 Canada

**Keywords:** *Cryptosporidium*, *Giardia*, Recovery, Water samples, USEPA Method 1623.1

## Abstract

**Objective:**

This study was designed to find a method to enhance the recovery of *Cryptosporidium* spp. and *Giardia* spp. parasites from water samples for research purposes compared to the results that can be achieved with USEPA Method 1623.1. Four different approaches were used to test water samples that were artificially spiked with parasites. The approaches were: (i) Method 1623.1 itself, (ii) elution of Method 1623.1 combined with microfiltration, (iii) an elution technique based on grinding the filter membrane in a blender before the eluent was concentrated by immunomagnetic separation, and (iv) the blender elution followed by microfiltration. Fluorescence microscopy was used to determine which approach led to the highest parasite recovery rates.

**Results:**

Method 1623.1 gave the best results for *Giardia,* while all four approaches were statistically equivalent for *Cryptosporidium*. We evaluated the costs and laboratory time requirements for each protocol to give readers a complete comparison of the methods tested. Elution of Method 1623.1 combined with microfiltration resulted in lower costs and less laboratory work time without compromising the recovery of the parasites.

**Supplementary Information:**

The online version contains supplementary material available at 10.1186/s13104-022-06118-9.

## Introduction

*Cryptosporidium* spp. and *Giardia* spp. are parasitic protozoa responsible for gastrointestinal illness in several animal species as well as in humans [1, 2]. One of the predominant means of dissemination of these parasites is the transmission of cysts, robust egg-like structures, through environmental water sources where they are consumed by new hosts [2]. To ensure the distribution of safe water for consumption, Canada mandates that water treatment plants reduce or inactivate 99.9% of *Cryptosporidium* spp. and *Giardia* spp. parasites in the water [3]. The United States requires 99% reduction of *Cryptosporidium* and 99.9% reduction of *Giardia* [4, 5]. Monitoring the presence of either of these organisms is crucial to validate the efficiency of the water treatment and to determinate the parasitic load of the source water entering the treatment plant. LeChevallier and collaborators analyzed 66 surface waters of the United States and of Canada, and found average concentrations of 0.04–66 *Giardia* cysts of per 100 L compared to 0.07–484 *Cryptosporidium* oocysts per 100 L [6]. In a document of 2009, World Health Organization stated that environmental waters worldwide can contain concentrations from 0.01 to 100 oocysts of *Cryptosporidium* per Litre [7].

Although several biomolecular protocols have been developed since the 1990s to detect these protozoa in environmental samples [8], the only actual standardized method to do it is United States Environmental Protection Agency (USEPA) Method 1623.1. Briefly, this method consists of filtering from 10 to 50 L of raw water with an EnviroChek HV filtration cartridge with a porosity of 1 µm. The biological material is eluted from the filter and parasites are concentrated by immunomagnetic separation (IMS). Then, their cells are enumerated by differential interference microscopy and fluorescence microscopy (with fluorescein isothiocyanate and 4′,6-diamidino-2-phenylindole to stain the cells). Although validated multiple times, this method has several weaknesses, such as common recovery rates for parasites of 30–50% and even lower [9–11], a laboratory-work time of several days, for a cost of about 1000 Canadian dollars (CAD) per sample analyzed (Ministère de l'Environnement et de la Lutte contre les changements climatiques, pers. comm.). Alternative elution techniques presented in previous studies gave variable recovery results from 9 to 58% for *Cryptosporidium* and 2 to 74% for *Giardia*. [12–18]. Therefore, we undertook experiments to test an alternative elution protocol combined either with IMS or microfiltration, to try to increase recovery rates of these parasites over the USEPA Method 1623.1 standardized steps. We also sought to shorten the procedures and reduce the costs associated with the analyses. Although none of the approaches tested in this study gave higher recoveries than the standardized method, some alternative protocols yielded statistically similar recovery rates, while reducing the time and costs required.

## Main text

### Methods

#### (Oo)cyst preparation and filtration

The spiking process was adapted from the matrix spike control described in USEPA Method 1623.1. To prepare artificially contaminated suspensions of oocysts of *Cryptosporidium* and cysts of *Giardia*, ColorSeeds (BioPoint Inc., Australia) containing 98–102 (oo)cysts per vial, which were inactivated by gamma ray exposure and permanently dyed with Texas Red stain, were used. Suspensions were prepared by adding a vial of ColorSeeds to 10 L of ultrapure water in a Cubitainer, plus the equivalent of 500 000 non-pathogenic bacterial cells belonging to the species *Bacillus cereus*, *Pseudomonas putida*, *Cupriavidus sp.,* and *Escherichia coli*, to mimic the microbial content of environmental water samples. The number of cells was determined by adjusting the optic density of the suspension with a standard of known concentration. The artificially contaminated samples were filtered with EnviroChek HV filtration cartridges (Pall Corporation, United States) with a peristaltic pump. Once the 10 L volume was done filtering, an additional 1 L of ultrapure water was added to the Cubitainer and shaken to collect potentially remaining adhered (oo)cysts. This volume was also filtered on the same cartridge. Cartridges were kept at 4 °C for a maximum of 24 h before processing.

#### Elution and centrifugation

**Fig. 1:**
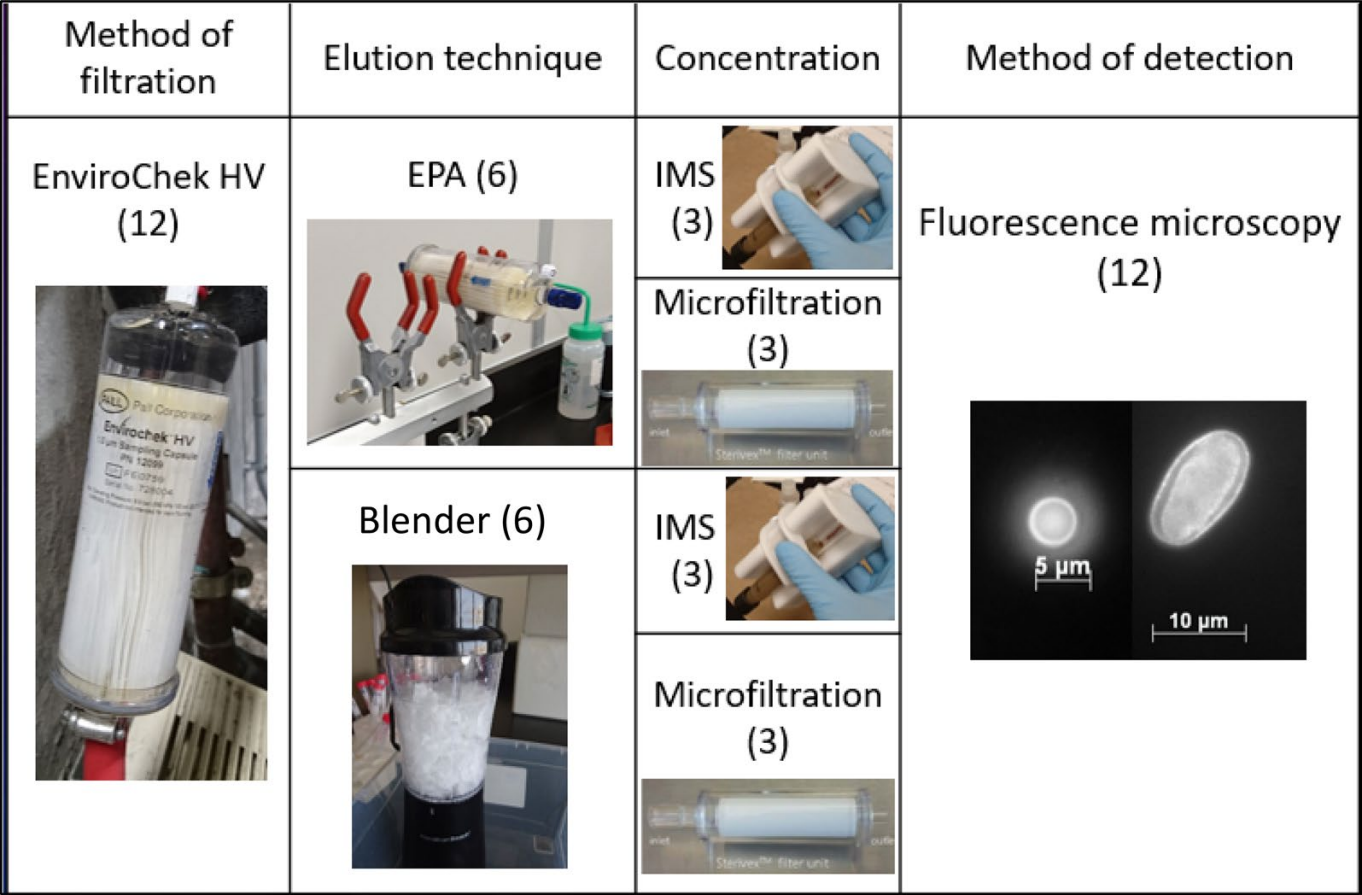
Summary of approaches tested and repartition of samples. EPA: elution protocol as described in U.S. EPA Method 1623.1; Blender: Alternative elution protocol consisting of opening the EnviroChek filtration cartridge, slicing the membrane surface and blending the pieces in a commercial blender; IMS: concentration by immunomagnetic separation according to the USEPA Method 1623.1; Microfiltration: concentration by filtrating the eluate with a Sterivex.™ filter of 0.45 µm of pore size

Samples were attributed to a combination of elution and concentration techniques, as illustrated in Fig. 1. Briefly, the twelve cartridges were split between two elution methods: standard USEPA Method 1623.1 [19], and an alternative elution protocol. The latter consists of opening the EnviroChek cartridge inside a laminar flow-hood cabinet with a pipe cutter, slicing the filtration membrane with a scalpel blade, and putting the fragments of the membrane in a commercial blender (Hamilton Beach, United States). The result of the cartridge opening can be seen at figure S1 (Additional file 1). The inside of the blender was coated with liquid silicone (SigmaCote, Sigma-Aldrich, Canada) to prevent the adherence of the (oo)cysts to the surfaces, as both parasites have hydrophilic surfaces [20]. Then, 250 mL of an elution solution containing Laureth-12 10%, Tris 1 M pH 7.4, EDTA 0.5 M pH 8.0, ultrapure water, and Antifoam A was added to the blender. The mixture was blended for 1 min. Then, liquid (with as few membrane pieces as possible) was collected in a centrifugation bottle. Its content was centrifuged for 30 min at 5855×*g* to collect the parasites in the pellet. This speed and duration were determined to be more efficient in pelleting parasites during preliminary tests with approximately 97% of oocysts pelleting.

#### Concentration of (oo)cysts

Products either from the USEPA 1623.1 standardized elution technique or from the alternative elution protocol were submitted to a concentration of the (oo)cysts into a smaller volume. To do so, two concentration techniques were compared to determine the efficiency of each, namely the IMS and an alternative microfiltration protocol. The IMS was performed according to USEPA 1623 protocol and using the Dynabeads™ GC-Combo kit (ThermoFisher Scientific, United States). The microfiltration was done by manually pumping the elution liquid into a sterile syringe and by pushing the solution into a Sterivex™ sterilizing filter of porosity of 0.45 µm. Then, biological material from the Sterivex™ was recovered following the extraction protocol described in [21], which includes opening the cartridge and cutting out the membrane. The membrane pieces were resuspended in 200 µL of PBS 1X buffer and vortexed one minute to allow the cells to be collected in the liquid.

#### Detection and enumeration of (oo)cysts

Concentrates either from the IMS or the microfiltration were then fixed on a microscope slide and stained with either EasyStain kit (BioPoint Inc., Australia) or Aqua-Glo™ G/C kit (Waterborne Inc., United States) following the manufacturer's instructions as required by the USEPA Method 1623.1. The slides were analyzed by fluorescence microscopy with a Zeiss Axio Observer Z1 microscope connected with an Axiocam MRm camera (Carl Zeiss, North York, ON, Canada) to enumerate the number of (oo)cysts collected. The techniques were compared by calculating the percentage of (oo)cysts collected by each one.

#### Statistical analysis

Each combination of steps was done in biological triplicate. Statistical analyses were made with the R package RCommandr version 3.5.0. The normality of the recovery values for each condition was determined by a Shapiro–Wilk test. Averages obtained for each condition and each parasite were then compared by the Kruskal–Wallis non-parametric test with a threshold of 5%. The data for the conditions applying to a normal distribution were compared together with an ANOVA test with a threshold of 5%.

### Results

**Fig. 2:**
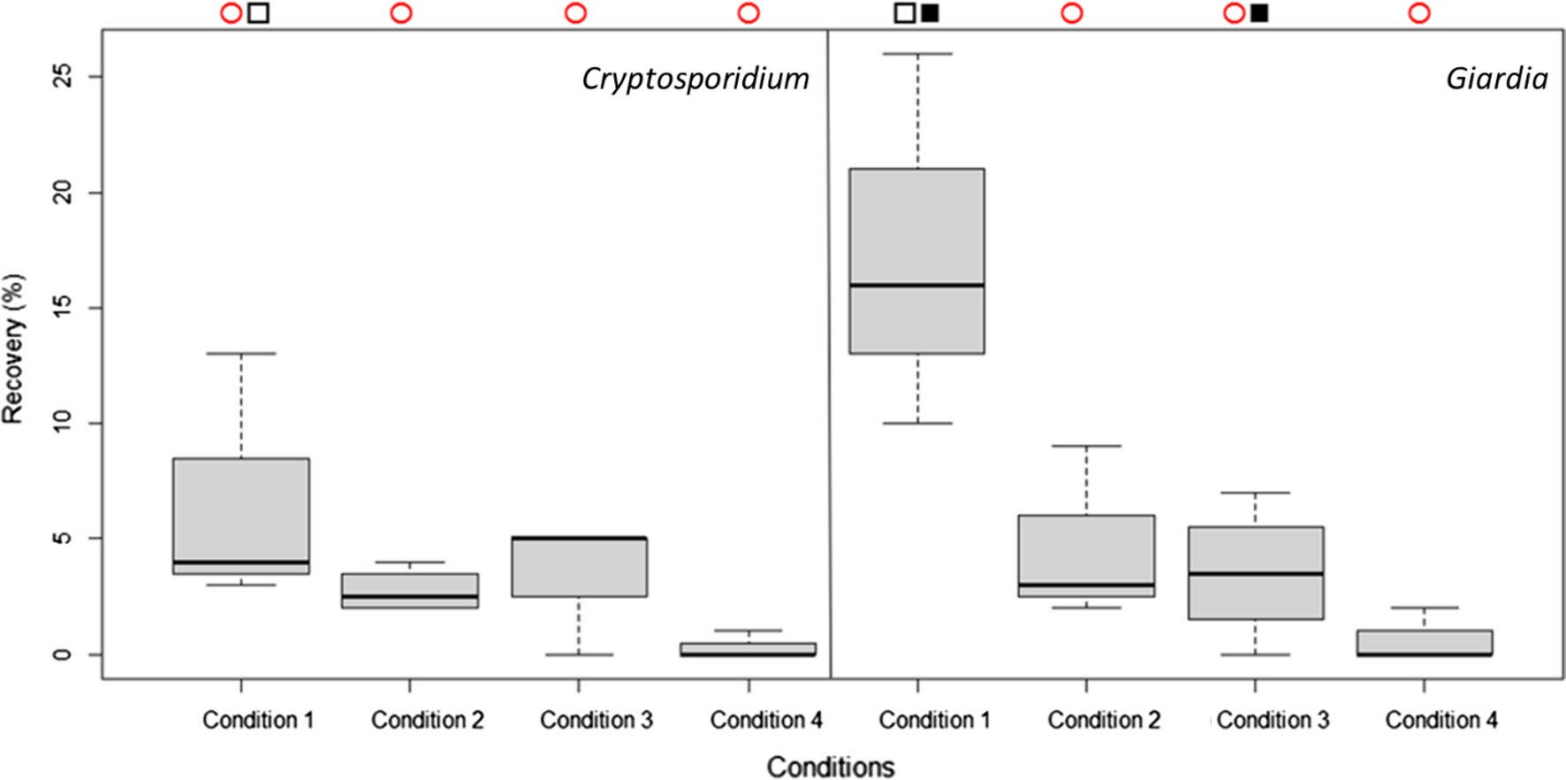
Recovery of *Cryptosporidium* and *Giardia* according to the combination of techniques applied. Condition 1 consists of the complete protocol according to USEPA Method 1623.1. Condition 2 is the combination of the elution according to the USEPA with the concentration by microfiltration. Condition 3 is the alternative elution protocol combined with the concentration by IMS. Condition 4 is the alternative elution protocol paired with the concentration by microfiltration. The symbol ■ states that the averages are statistically significantly different with a threshold of 5%. The symbol □ indicates that the averages are non-statistically significantly different with a threshold of 5%. The symbol ○ means that the medians are non-statistically significantly different with a threshold of 5%

Figure 2 presents the results obtained with the various combinations of techniques. Although none of these alternative approaches gave significantly superior recovery rates to USEPA Method 1623.1, equivalent results were obtained in the case of *Cryptosporidium* results. It was determined that the medians of the data generated with the four approaches were not statistically different for this parasite. For *Giardia*, the USEPA Method 1623.1 produced recovery rates with a median statistically significantly higher than the three other approaches tested. Even if the distribution of data could look otherwise, averages of the data obtained for *Cryptosporidium* and *Giardia* with the USEPA Method 1623.1 were not statistically different. But this global behavior of higher recoveries for *Giardia* than for *Cryptosporidium* has been seen in previous studies as well [17, 22, 23]. Only the comparison of data from the USEPA protocol for *Giardia* and the blender elution followed by IMS gave statistically significantly different averages, with the USEPA method giving higher values.

The costs and the laboratory work time required for each approach are summarized in Table 1 and detailed. Some of the alternative approaches allow a gain of time of a few hours of laboratory work. The costs can be significantly reduced from 100 to 650 CAD depending on the method chosen compared to the USEPA Method 1623.1. It could be advantageous especially in the case of *Cryptosporidium* where all techniques generated non-statistically significant different results. Despite the decrease in costs, the alternative elution paired with the microfiltration does not seem to be the best approach to adopt, given the low recoveries obtained. However, USEPA elution with microfiltration appears to be the most advantageous combination of alternative approaches regarding the time gain and the decrease of costs for a similar recovery. This is particularly promising in a research context.

**Table 1:** Costs and time required per sample for each combination of techniques tested in this study

Approach	Costs (in CAD) **	Time required *
USEPA Method 1623.1	1000$	6 h
USEPA elution with microfiltration	570$	4 h
Alternative elution with IMS	900$	5 h
Alternative elution with microfiltration	350$	3 h

^*^The time here excludes preparation of microscopic slides and observation in microscopy (approximately 10 h) since the duration of these steps is identical for each approach

^**^Costs calculated include consumables and equipment only. The cost of equipment was amortized on a hundred samples. The detailed analysis of the costs is presented in the Additional file 1 (Table S1).

## Limitations

Some limitations in the approaches tested in this study are worth mentioning. First, our inexperience with the USEPA Method 1623.1 protocol likely may have contributed to the relatively low recovery rates. However, low recovery rates were also reported in the past by interlaboratory validation assays led by the USEPA [11], and our recovery rates align with those. With experience and practice, higher recoveries might be expected. Typically, only well-trained personnel adept at obtaining higher recoveries are allowed to analyse samples from clients in environmental analysis laboratories, while the present study was done in an academic research context.

Second, with the alternative elution protocol tested during this study, it was not possible to physically retrieve the entire filtration membrane from the EnviroChek HV cartridge following its opening with a pipe cutter. Approximately 30% of the filtration surface remained covered by the polycarbonate housing despite all our efforts to cut the cartridge closer to the extremity (Additional file 1, Figure S1). Therefore, the parasites stuck to that portion of the filtration surface could not be collected with this approach.

Third, although the concentration of parasites by IMS is expected to remove all other cells that do not belong to either *Cryptosporidium* or *Giardia* genera, our experiments showed that does not always occur. Many bacteria cells could also be seen (Additional file 1, Figure S2). Our experiments were done by artificially spiking 10 L of water with 500 000 bacterial cells to mimic contaminated environmental water. However, some raw water samples may contain even higher concentrations of bacteria. For example, the River Ruhr in Germany, which was studied by Strathmann et al. [24]*,* contained about 3.4 × 10^6^ total cells per mL (more than 50 000 times more bacteria than in our own samples). We conclude that the problem of interfering bacteria carried over during the IMS could be quite cumbersome for the analysis of some environmental samples and could interfere with the analysis. IMS beads can also confuse *Cryptosporidium* oocyst detection if they are carried over in the sample until the microscopic examination (see Additional file 1, figure S3 for comparison).

Finally, centrifugation is a major issue to consider in this protocol to improve parasite recovery. The USEPA Method 1623.1’s centrifugation step is at 1500 × *g* for 15 min. In a previous study, this centrifugation caused a loss of 8 to 14% of cells when compared with the same sample composition submitted only to IMS and fluorescence microscopy [25]. Lechevallier et al*.* [26] found that higher centrifugation speed helped to recover more (oo)cysts; therefore, to increase the proportion of (oo)cysts collected, we chose a centrifugation speed of 5855 × *g* for 30 min following our alternative elution technique. Since Lechevallier et al*.* (26) demonstrated that even a speed of 17 300 × *g* would not disrupt them, it is unlikely that the centrifugation applied here would have broken them. We did not evaluate the loss of parasites at each step in the present study.

## Supplementary Information

**Additional file 1:**. **Figure S1.** Photograph of the EnviroChek HV filtration cartridge following opening with a pipecutter during the alternative elution protocol. **Figure S2.** Picture of bacteria carried over by the immunomagnetic separation. **Figure S3.**. Comparison of a *Cryptosporidium* oocyst (A) with beads from the IMS beads (B).**Table S1.** Detailed evaluation of the costs associated with the techniques presented in this article.

## Data Availability

The datasets used and/or analyzed during the current study are available from the corresponding author on a reasonable request.

## Abbreviations

IMS: Concentration by immunomagnetic separation
USEPA: United States Environmental Protection Agency
