# Supplementary material for: Working toward improved monitoring of Cryptosporidium and Giardia (oo)cysts in water samples: testing alternatives to elution and immunomagnetic separation from USEPA Method 1623.1
Source: BMC Res Notes. 2022 Jul 15;15:254. doi: 10.1186/s13104-022-06118-9 (PMC9284717; doi:10.1186/s13104-022-06118-9)
Supplement: Supplementary file 1 — Additional file 1:. Figure S1. Photograph of the EnviroChek HV filtration cartridge following opening with a pipecutter during the alternative elution protocol. Figure S2. Picture of bacteria carried over by the immunomagnetic separation. Figure S3.. Comparison of a Cryptosporidium oocyst (A) with beads from the IMS beads (B).Table S1. Detailed evaluation of the costs associated with the techniques presented in this article. [file 13104_2022_6118_MOESM1_ESM.docx]

# **Attachment 1**

# **Working toward improved monitoring of *Cryptosporidium* and *Giardia* (oo)cysts in water samples: testing alternatives to elution and immunomagnetic separation from U.S. EPA Method 1623.1**

Authors:

Marie-Stéphanie Fradette ^1,2,3,4^* (marie-stephanie.fradette.1@ulaval.ca),

Steve J. Charette ^1,2,3^ (Steve.Charette@bcm.ulaval.ca)

1. Institut de Biologie intégrative et des systèmes (IBIS), Laval University, 1030, avenue de la Médecine, Québec City, Québec, Canada, G1V 0A6

2. Département de Biochimie, de Microbiologie et de Bio-informatique, Faculté des Sciences et Génie, Laval University, 1045, avenue de la Médecine, Québec City, Québec, Canada, G1V 0A6

3. Centre de Recherche de l’Institut Universitaire de Cardiologie et de Pneumologie de Québec, 2725, chemin Sainte-Foy, Québec City, Québec, Canada, G1V 4G5

4. Centre de Recherche en Aménagement et Développement du territoire (CRAD), Laval University, 2325, allée des Bibliothèques, Québec City, Québec, Canada, G1V 0A6

*Correspondence: Marie-Stéphanie Fradette (marie-stephanie.fradette.1@ulaval.ca)


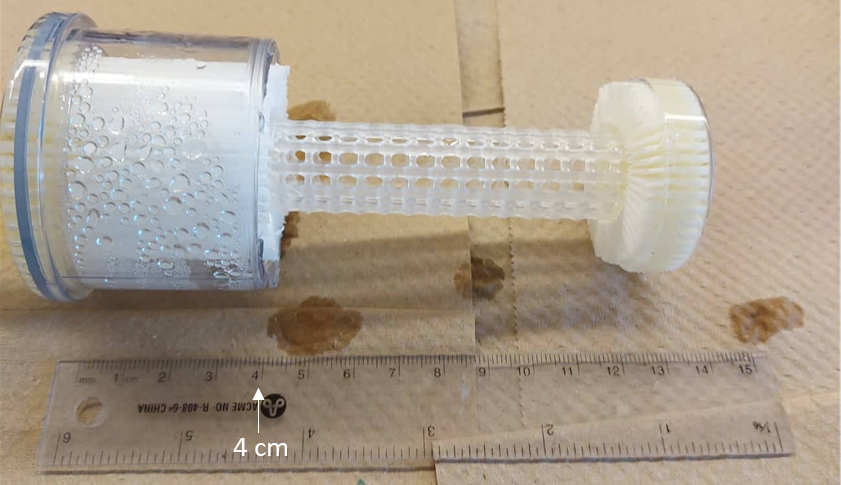

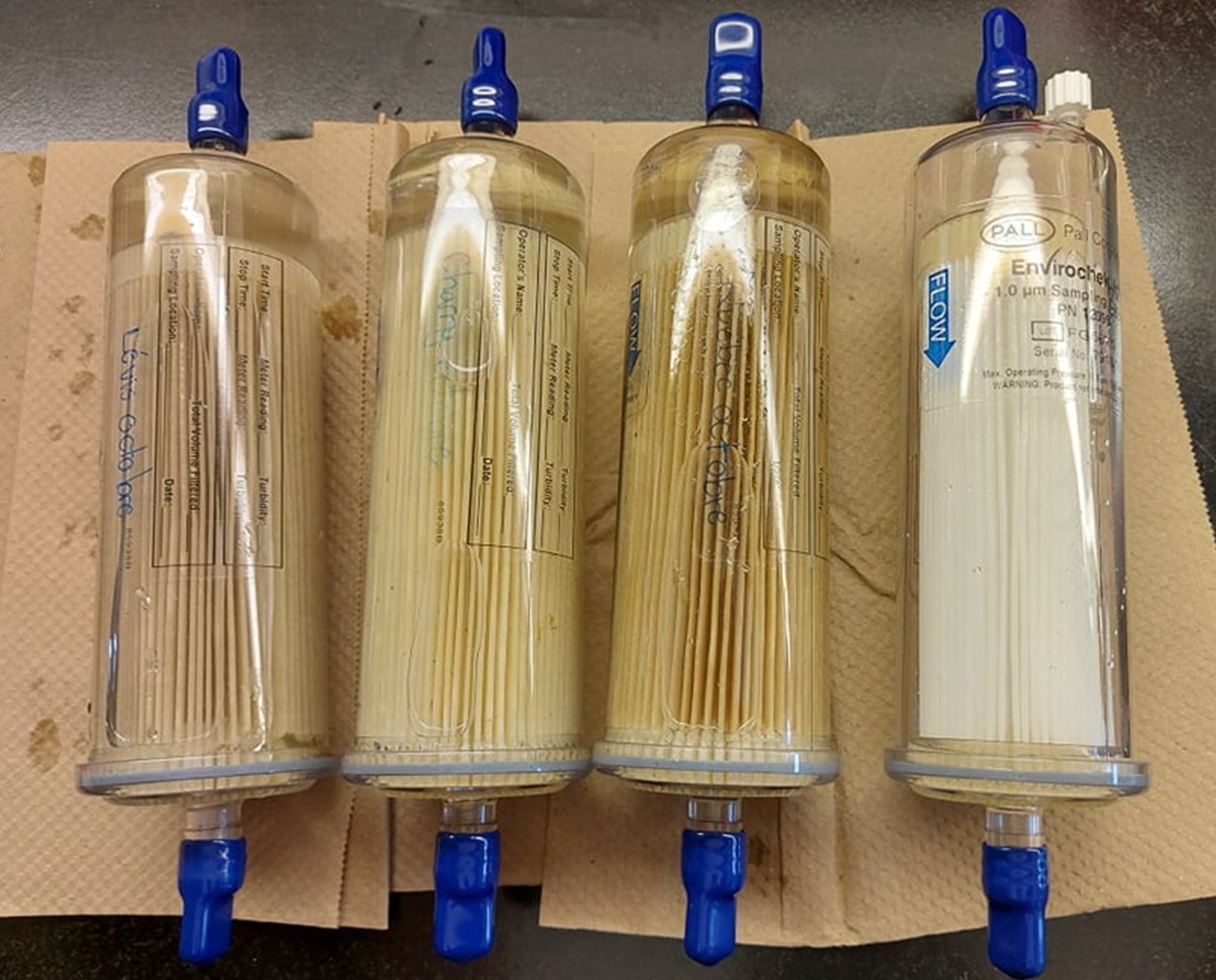


B

A

**Figure S1. Photograph of the EnviroChek HV filtration cartridge following opening with a pipe-cutter during the alternative elution protocol.** A. Before the cut B. After the cut. Note that a portion at 4 cm could not be retrieved because of the presence of the plastic edge at the extremity that prevented the pipe-cutter from cutting further.


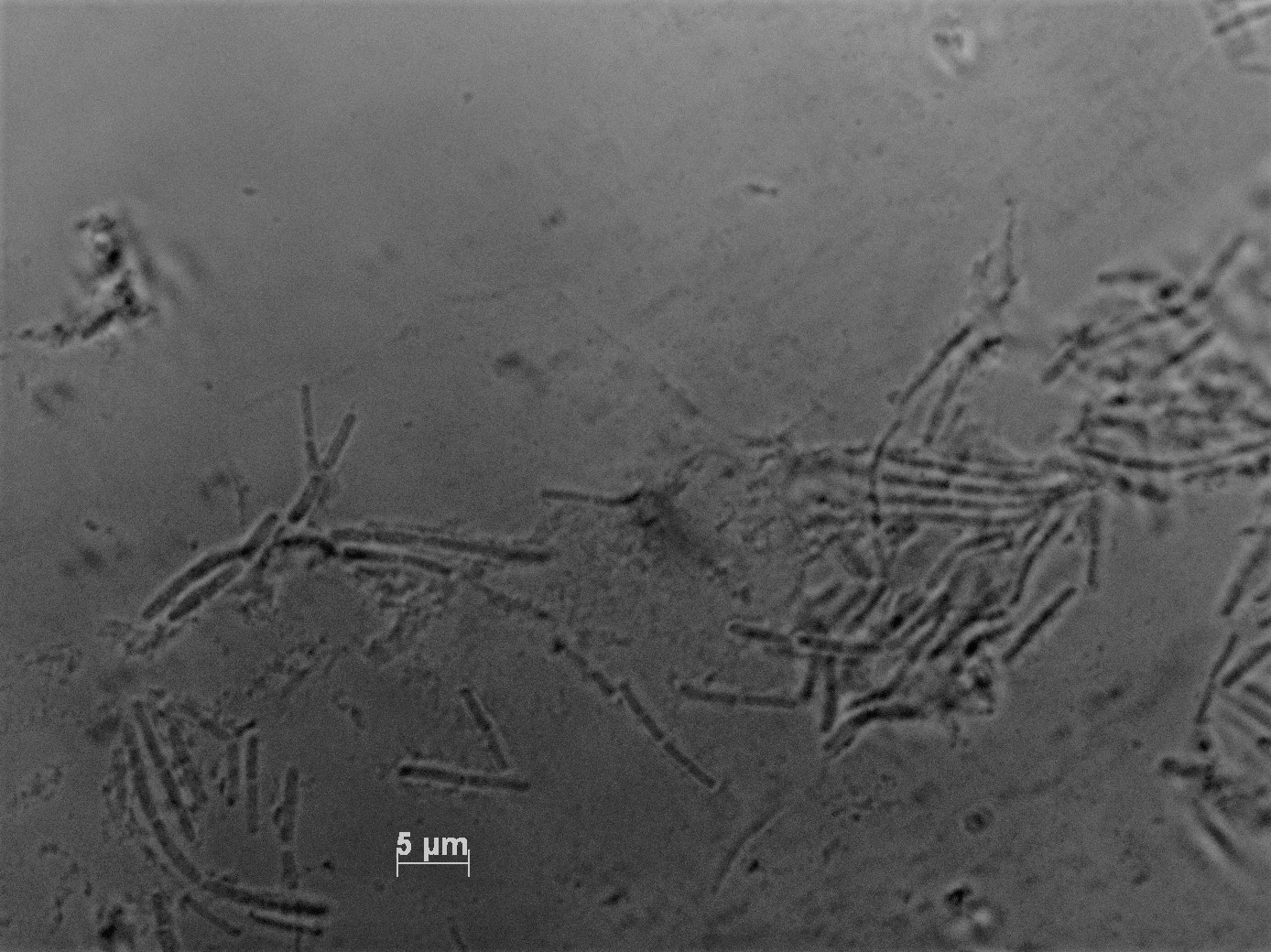
**Figure S2. Picture of bacteria carried over by the immunomagnetic separation.** Bacteria belonging to the genera *Bacillus cereus*, *Pseudomonas putida*, *Cupriavidus sp.* and *Escherichia coli* were added to the artificially contaminated samples. Long bacilli of approximately 5 µm, likely *B. cereus* or *Pseudomonas putida,* can be seen in majority. Coccobacilli such as *Cupriavidus* and/or *E. coli* can also be observed among the other cells. This phenomenon could be seen in all samples, for all the elution techniques we used.







B

A

**Figure S3. Comparison of a *Cryptosporidium* oocyst (A) with beads from the IMS beads (B).** The scale bar is the same for both pictures. Beads are spherical with a diameter of 5 µm, which is about the same size and shape as oocysts. Oocysts display a bright luminous halo on their periphery, while beads do not produce this halo. Under the red filter on the fluorescent microscope, beads appear red like ColorSeeds, yet a bit dimmer than oocysts.

**Table S1. Detailed evaluation of the costs associated with the techniques presented in this article.**

| Equipment/consumable | Cost per sample (CAD) |
| --- | --- |
| EnviroChek HV cartridge | 138.00 |
| U.S. EPA elution | 404.05 |
| Alternative elution | 191.58 |
| IMS | 554.33 |
| Microfiltration | 10.96 |
| Slide preparation | 11.36 |
